# Challenges in Diagnosing Foreign Body Aspiration in Children

**DOI:** 10.7759/cureus.21519

**Published:** 2022-01-23

**Authors:** Pei Li Goh, Eng Haw Lim, Davina Stasia Hui Ming Teo, Mohd Eksan Sairin

**Affiliations:** 1 Otorhinolaryngology - Head and Neck Surgery (ORL-HNS), Hospital Miri, Sarawak, MYS

**Keywords:** bronchopneumonia, respiratory distress, children, bronchoscopy, foreign body aspiration

## Abstract

Foreign body aspiration in children is a medical emergency that is associated with significant morbidity and requires medical intervention. The variability of clinical presentations results in delayed diagnosis and treatment. Hence, a high index of clinical suspicion and a thorough examination is needed to expedite the management. We report the case of a toddler girl who aspirated a piece of peanut and was diagnosed as having bronchopneumonia upon presentation with respiratory symptoms for six days. Rigid bronchoscopy was performed and the foreign body was removed successfully without any complications. We aim to highlight the importance of considering the diagnosis of airway foreign body in children with unexplained respiratory distress in our case study.

## Introduction

Foreign body (FB) aspiration in children is a medical emergency and can be life-threatening. It is frequently seen and is commonly classified into organic or inorganic FB aspiration. The most commonly aspirated organic FB found in the Malaysian pediatric group is peanuts [1]. FB aspiration results in high morbidity, ranging from 10-20% worldwide, and accounts for 7% of accidental deaths in children under four [2]. The pediatric population is more susceptible to FB aspiration due to a lack of molar teeth, underdeveloped swallowing coordination, and the tendency to talk or play while ingesting [3]. The presenting signs and symptoms in this group of patients are highly variable and may result in severe sequelae ranging from choking, respiratory distress, cyanosis, and asphyxia, which may mimic other respiratory illnesses and infections [1,4]. The gold standard for the treatment of aspirated FB is rigid bronchoscopy with forceps removal. Flexible bronchoscopy is sometimes used to localize the FB prior to removal by rigid bronchoscopy and to remove FB located peripherally [1,5-6]. Thorough clinical history and examination are crucial in the diagnosis of airway FB as 85% of the cases can be detected during the first physician encounter, whilst 15% of cases may go unnoticed and result in complications such as pneumonia and atelectasis [7]. Here, we present a case of delayed diagnosis of FB aspiration in a child.

## Case presentation

A previously healthy, two-year seven-month-old girl was admitted to the pediatrics unit for rapid breathing of six days duration. She had a history of choking episodes following peanut ingestion prior to admission. It was associated with brief cyanosis, cough with post tussive vomiting, noisy breathing, hoarseness, fever, and poor oral intake. Her symptoms were not induced by coryza symptoms. She initially presented to a district hospital on day one of illness and was treated as acute tonsillitis and discharged with oral antibiotics. Subsequently, on day four of illness, she developed one episode of hemoptysis and was admitted to the district hospital with bronchopneumonia and started on intravenous ampicillin. Her condition further deteriorated and required an oxygen supplement prior to transfer to our tertiary center for further management.

**Figure 1:**
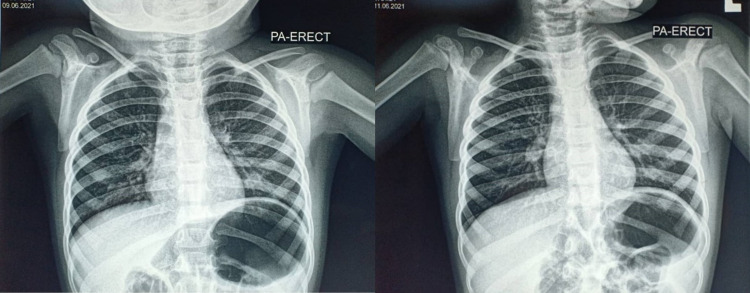
Chest X-ray (PA erect view) taken: clear lung fields and no foreign body seen PA: posterior-anterior

Physical examination reviewed a well-developed girl with hoarseness but no stridor. Her peripheral capillary oxygen saturation (SpO2) was 100% on 2 liters of oxygen delivered via nasal cannula. Oral examination revealed bilateral tonsils grade 3 with no obvious FB. Auscultation of the lungs showed equal air entry with transmitted sounds bilaterally. Other systemic examinations were unremarkable. The bedside flexible laryngoscopy did not review FB or any abnormalities. Chest X-rays showed no evidence of pneumonia and no dense FB within (Figure 1). Her blood investigations reviewed leukocytosis of 17,700.

**Figure 2:**
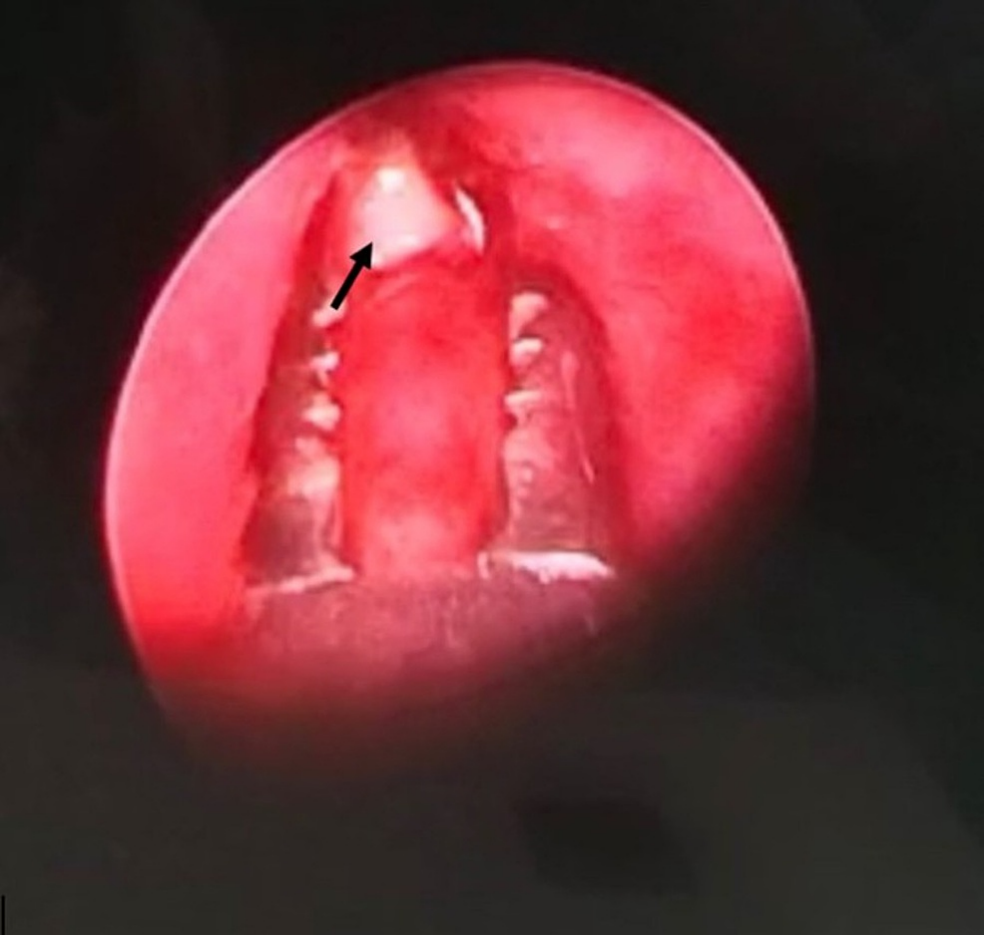
Endoscopic view of the foreign body (arrow) seen in the left main bronchus during rigid bronchoscopy

**Figure 3:**
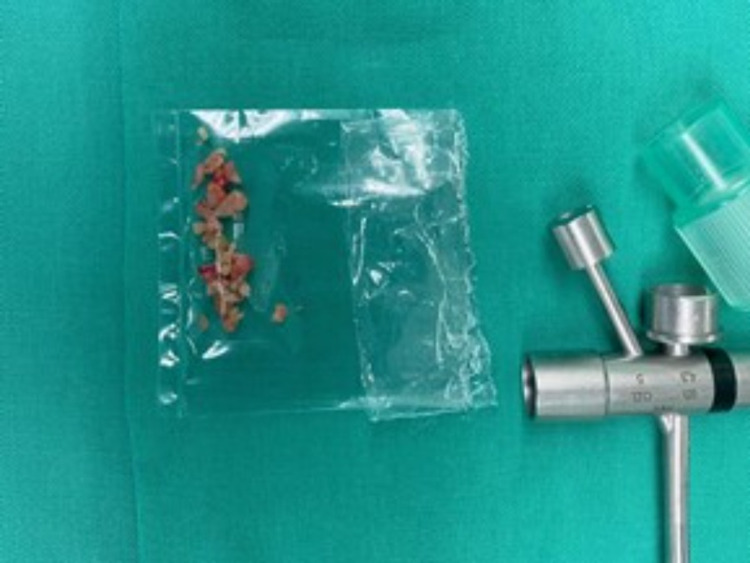
Pieces of peanuts retrieved from the left main bronchus

Direct laryngoscopy and rigid bronchoscopy were done under general anesthesia. She was intubated with an endotracheal tube of size 4.5. General anesthesia was maintained by total intravenous anesthesia with fentanyl and propofol followed by targeted controlled infusions of remifentanil and propofol. A piece of peanut was visualized in the left bronchus, 1 cm from carina (Figure 2). The peanut was removed in piecemeal using optical bronchial foreign body forceps, as it was friable and broken into pieces (Figure 3). Postoperatively, it was uneventful, and she weaned off supplemental oxygen. She was discharged well on postoperative day two.

## Discussion

Foreign body aspiration is not uncommon, especially in the pediatric age group. Pneumonia is the leading cause of illness and death worldwide in children under the age of five [7]. The patient may present with a history of FB ingestion and remains asymptomatic especially when the FB is lodged at a particular area until further complications develop in severe sequelae [6,8]. In our patient, the co-infection with upper respiratory tract infection, acute tonsillitis may cloud the clinician’s judgment in diagnosing an airway FB initially. This case study aims to emphasize the importance and need to actively exclude airway FB among pediatric groups, especially in those under three years of age, with a history of choking episodes following peanut ingestion and unexplained respiratory distress.

Sahadan et al. reported in their review that the peak incidence was under the age of three due to the habit of putting objects into their mouth, crying, or playing with objects inside their mouth causing accidental ingestion and aspiration [5]. Goyal et al. reviewed that the most common features in patients who presented within a week were tachypnea (58.8%), wheeze (47.05%), and wet cough (41.1%). Fever (65%) and dry cough (45%) were the main complaints in those presented after a week. A delayed presentation was mostly due to aspiration unnoticed by caretakers, poor clinical history, and delayed referral.

Chest radiographs are usually utilized as the initial diagnostic modality in a suspected case of FB aspiration. The FB may be identified as either radio-opaque or radiolucent, which is seen in most objects. Radiolucent objects are hardly picked up on plain radiographs [1]. Hence, a computed tomography scan is better in these cases [4]. Other radiographic features include hyperinflation, consolidation, lung collapse, atelectasis, pneumothorax, and tracheal or mediastinal shift [1,9]. As seen in our patient, some cases may present with normal chest X-rays, as most FB are organic and not visible on X-ray [10].

Cassol et al. reported in their earlier publication that the most frequent initial clinical diagnoses given were asthma, pneumonia, laryngitis, and bronchiolitis [11]. This may result in delayed diagnosis and further airway insult. Rigid bronchoscopy remains the gold standard for the removal of an aspirated FB in children and especially in those who present with acute respiratory distress [8]. The most common site where FBs are found was in the right bronchus, as it is anatomically shorter and wider. Aspiration to a specific location depends upon the position the child was in when aspiration occurred. The right lower lung lobe is the most common site, as it is larger and more vertical. Bilateral lower lung lobe infiltrates commonly occur in patients who aspirate while standing, whereas lying in the left lateral decubitus position is more likely to cause left-sided infiltrates. Alcoholics who aspirate while in a prone position may result in right upper lobe infiltrates. In our case, it was also lodged in the left main bronchus.

Organic objects, such as nuts, may break up into smaller pieces and expand due to the absorption of water. This feature may subject the patient to develop complete airway obstruction from a partially obstructed airway. Besides, smaller pieces may lodge into distant airways and be difficult to remove [1]. If there is clinical suspicion of an aspirated FB in children, even with a negative chest X-ray, prompt removal with bronchoscopy of the FB is required [6]. Immediate intervention is needed in handling such cases.

## Conclusions

Foreign body aspiration is an emergency and should be suspected in children, especially with a history of choking. It is important for all healthcare personnel to be vigilant and aware of the potential hazardous sequelae that follow. A high index of suspicion is required to establish the diagnosis without delaying the definitive treatment and to avoid misdiagnosis.
